# A comprehensive review on Ellagic acid in breast cancer treatment: From cellular effects to molecular mechanisms of action

**DOI:** 10.1002/fsn3.3699

**Published:** 2023-09-18

**Authors:** Maryam Golmohammadi, Mohammad Yasin Zamanian, Sahbanathul Missiriya Jalal, Sara Abdalrazzaq M. Noraldeen, Andrés Alexis Ramírez‐Coronel, Khulood H. Oudaha, Rasha Fadhel Obaid, Abbas F. Almulla, Gholamreza Bazmandegan, Zahra Kamiab

**Affiliations:** ^1^ School of Medicine Shahid Beheshti University of Medical Sciences Tehran Iran; ^2^ Department of Physiology, School of Medicine Hamadan University of Medical Sciences Hamadan Iran; ^3^ Department of Pharmacology and Toxicology, School of Pharmacy Hamadan University of Medical Sciences Hamadan Iran; ^4^ Nursing, College of Applied Medical Sciences King Faisal University Al‐Ahsa Saudi Arabia; ^5^ Department of Medical Laboratory Technology University of Tabuk Tabuk Saudi Arabia; ^6^ Research Group in Educational Statistics National University of Education (UNAE) Azogues Ecuador; ^7^ Epidemiology and Biostatistics Research Group CES University Medellín Colombia; ^8^ Pharmaceutical Chemistry Department, College of Pharmacy Al‐Ayen University Thi‐Oar Iraq; ^9^ Department of Biomedical Engineering Al‐Mustaqbal University College Babylon Iraq; ^10^ Department of Medical Laboratory Technology, College of Medical Technology Islamic University Najaf Iraq; ^11^ Physiology‐Pharmacology Research Center, Research Institute of Basic Medical Sciences Rafsanjan University of Medical Sciences Rafsanjan Iran; ^12^ Department of Physiology and Pharmacology, School of Medicine Rafsanjan University of Medical Sciences Rafsanjan Iran; ^13^ Clinical Research Development Unit, Ali‐Ibn Abi‐Talib Hospital Rafsanjan University of Medical Sciences Rafsanjan Iran; ^14^ Department of Community Medicine, School of Medicine Rafsanjan University of Medical Sciences Rafsanjan Iran

**Keywords:** anti‐tumor activity, breast cancer, Ellagic acid, signaling pathways

## Abstract

Globally, breast cancer (BC) is the leading cause of cancer‐related deaths in women. Hence, developing a therapeutic plan to overcome the disease is crucial. Numerous factors such as endogenous hormones and environmental factors may play a role in the pathophysiology of BC. Regarding the multi‐modality treatment of BC, natural compounds like ellagic acid (EA) received has received increased interest in antitumor efficacy with lower adverse effects. Based on the results of this comprehensive review, EA has multiple effects on BC cells including (1) suppresses the growth of BC cells by arresting the cell cycle in the G0/G1 phase, (2) suppresses migration, invasion, and metastatic, (3) stimulates apoptosis in MCF‐7 cells via TGF‐β/Smad3 signaling axis, (4) inhibits CDK6 that is important in cell cycle regulation, (5) binds to ACTN4 and induces its degradation via the ubiquitin‐proteasome pathway, inducing decreased cell motility and invasion in BC cells, (6) inhibits the PI3K/AKT pathway, and (7) inhibits angiogenesis‐associated activities including proliferation (reduces VEGFR‐2 tyrosine kinase activity). In conclusion, EA exhibits anticancer activity through various molecular mechanisms that influence key cellular processes like apoptosis, cell cycle, angiogenesis, and metastasis in BC. However, further researches are essential to fully elucidate its molecular targets and implications for clinical applications.

## Graphical abstract

Ellagic acid is a naturally occurring polyphenolic compound found in various fruits and berries, known for its strong antioxidant and anticancer properties. It has been studied for its potential role in the prevention and treatment of cancer, including breast cancer.

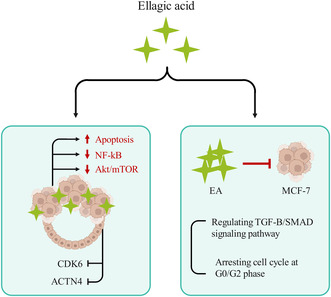

## 1 INTRODUCTION

Breast cancer (BC) is the second most prevalent cancer in women and has an alarming negative impact on societies specifically on the United States and Asian populations (Hammad et al., 2021; Landeros‐Martínez et al., 2016). Based on recent reports, an estimated 685,000 female death were due to BC worldwide which are responsible for about 16% of all cancer deaths (Chiodo et al., 2021; Zhu et al., 2021). BC five‐year survival rates vary according to the tumor stages, which are 99% for local and 27% for advanced breast malignancies (Mekkawy et al., 2022). The breast mass often originates from ducts or lobules and could be benign or aggressive, as well as spreading to the lymph nodes and invading other body tissues (Watkins, 2019).

Numerous risk factors are responsible for BC, including menopause, endogenous hormones, and oral contraception. Moreover, environmental factors, lifestyle, and family history have been identified to be accounted for developing breast mass (Key et al., 2001; Zamanian, Golmohammadi, Alalak, et al., 2023; Zamanian, Golmohammadi, Nili‐Ahmadabadi, et al., 2023). In recent decades, advanced therapy approaches and early identification through mammography lead to better prognoses and higher health outcomes (Ahmad, 2019). Although the etiology of BC is not entirely clear, more evidence showed that estrogen has an essential role in the proliferation and survival of malignant breast epithelial cells via binding to estrogen receptors (Hart et al., 2015; Li et al., 2010). According to receptor‐mediated breast carcinogenesis, estrogen exerts its effect through the estrogen‐receptor alpha (ERα), which induces cell growth and mutations following DNA replication errors and causes BC (Yue et al., 2010).

Generally, BC requires multi‐modality treatment including surgery, chemotherapy, radiotherapy, and hormone therapy (Zhu et al., 2021). Changes in socioeconomic status and risk factors lead to an increase in the incidence of BC despite technical advances in its diagnosis and management (Omran, 2005). BC is classified based on its tumor markers as well as histological and molecular types (Tang et al., 2016). ER‐positive is the most prevalent subtype of BC, so medication with tamoxifen, fulvestrant, and letrozole could block estrogen activity and prevent the estrogen‐dependent development of the majority of breast malignant tumors (Fabian, 2007; Llombart‐Cussac et al., 2021). Tamoxifen is a selective ER modifier that reduces the BC recurrence rate to 40%–50% and showed clinically profound impacts in lowering death rates (Karn, Jha, Shrestha, Acharya, et al., 2010). After attaching to the ER, it forms a nuclear compound that suppresses ER gene transcription (Binkhorst et al., 2012; Yen et al., 2022). Despite numerous therapeutic methods, BC is still a significant public health concern. Moreover, due to pharmacological adverse effects and radiation load lower compliance, finding new therapeutic anticancer agents is essential (Karn, Jha, Shrestha, & Poudel, 2010; Miele et al., 2009). In this regard, several bioactive compounds have been investigated to have some anticancer and cytotoxic activities (Yoganathan et al., 2021). Furthermore, these compounds could be adjuvant treatments that boost antitumor efficacy as well as lower the risks of other medications' side effects (Carrillo‐Navas et al., 2012; Goyal et al., 2022). Some research also revealed that plant secondary metabolites like polyphenols had excellent chemotherapeutic activities (Butler et al., 2004; Craig & Stitzel, 2004).

**FIGURE 1:**
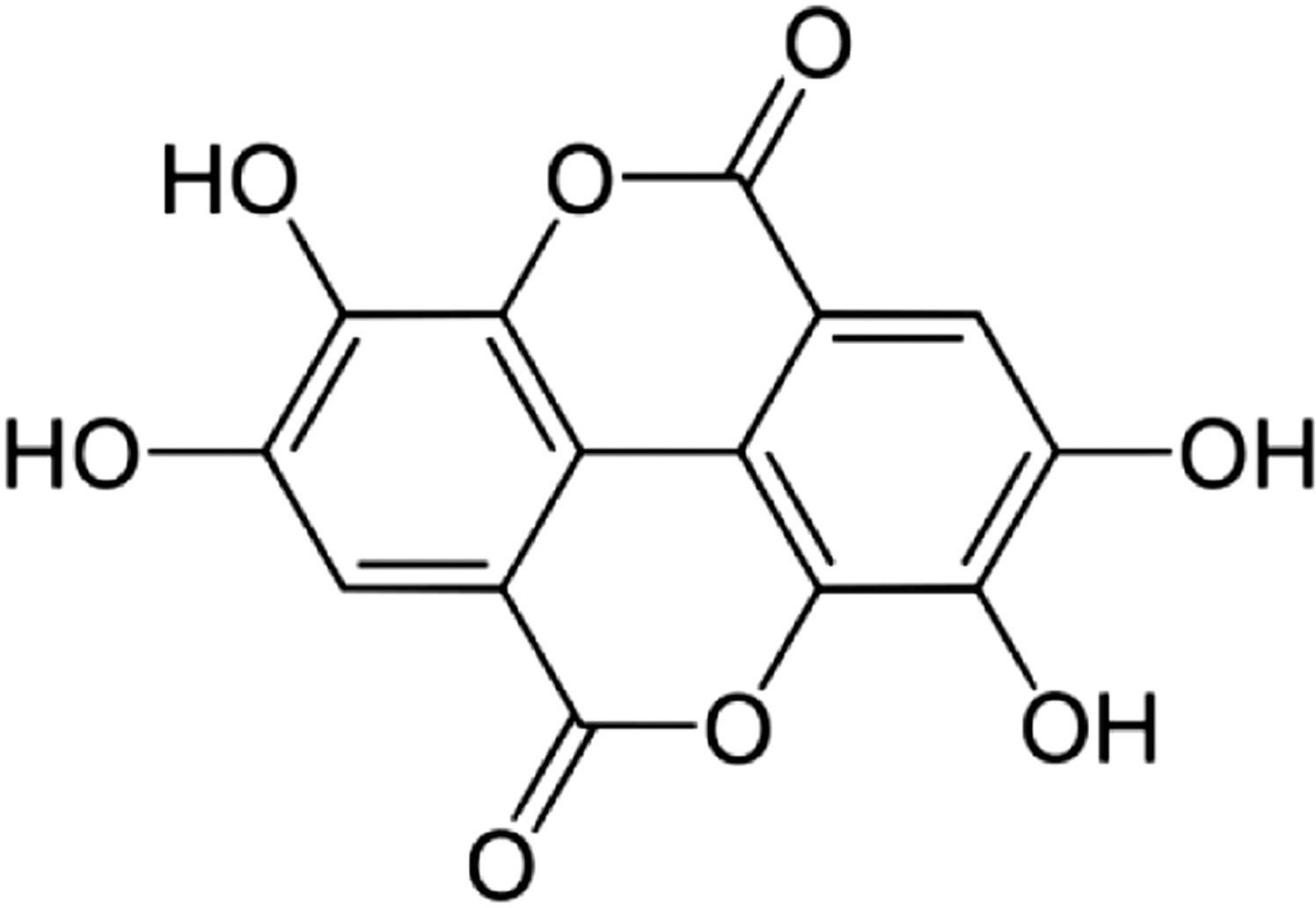
Chemical structure of Ellagic acid (Naraki et al., 2022; Xie et al., 2019).

Ellagic acid (EA, Figure 1), a nature‐derived polyphenolic substance, is found in many fruits, seeds, and nuts including pomegranates, black raspberries, strawberries, raspberries, almonds, and walnuts (Amakura et al., 2000; Cheshomi et al., 2022; Constantinou et al., 1995; Zhu et al., 2022). EA is a chromene‐dione derivative (C_14_H_6_O_8_) and can exist in a variety of forms, such as free, glycosylated forms, and combined forms ellagitannins (ETs) (Amakura et al., 2000; Ceci et al., 2018; Constantinou et al., 1995; Zhang, Zhao, et al., 2014). EA can enhance antioxidant enzyme activity, prevent lipid peroxidation, and decrease reactive oxygen species (ROS) generation (Derosa et al., 2016; Ríos et al., 2018). EA possesses antioxidant activity and anticancer properties that prevent angiogenesis, migration, and dissemination in different cancers like colorectal, pancreatic, breast, and bladder malignancy (Badr‐Eldin et al., 2022; Ceci et al., 2018; Goyal et al., 2022; Kim et al., 2021; Serretta et al., 2022). As regards, investigations detected that EA has inhibitory activity on BC growth through several pathways including apoptosis augmentation, cell cycle arrest, tumor angiogenesis suppression, antioxidant activity, and estrogen receptor gene regulation (Ahire et al., 2017; Kaur et al., 2021; Pirzadeh‐Naeeni et al., 2020). Yousuf et al. reported that EA treatment decreased the expression of cyclin‐dependent kinase 6 (CDK6), inhibited cell proliferation, and induced apoptosis in the BC cells (Yousuf et al., 2020). Additionally, EA notably reduced the number of colonies and caused apoptosis in BC cells (Yousuf et al., 2020).

The TGF‐β/Smads pathway is a well‐known signaling axis responsible for multiple physiological processes, like cell growth, differentiation, and apoptosis (Liu et al., 2019; Wang, Sun, et al., 2012). Chen et al. reported that EA inhibited the growth of BC cells by arresting the cell cycle and inhibiting proliferation, and that cyclins (cyclin A2 and cyclin E2) were downregulated in EA‐treated MCF‐7 cells, while CDK inhibitors (p21Cip1, p15, and p19) were overexpressed (Chen et al., 2015). Additionally, they found that ellagic acid induced apoptosis in MCF‐7 cells through the TGF‐β/Smad3 signaling axis (Chen et al., 2015). Besides, EA could enhance the efficacy of radiation and chemotherapy (Ceci et al., 2018).

Despite the clinical advancement of new therapies, BC is still a significant public health concern. The major objective of adjuvant treatment strategies in BC is to enhance patient life expectancy and quality of life (An & Hu, 2022). In terms of offering a viable solution for the management of BC, we intend to thoroughly outline the molecular and cellular mechanisms of EA and its anti‐oxidative protective role in BC in the present review.

## 2 OVERVIEW OF ELLAGIC ACID

EA, a phenol substance, was found in 1831. Since EA's emergence, more studies depicted its anti‐tumor and antioxidant activities (Law, 1922). EA as a thermostable and poor‐water soluble compound could be present in a variety of forms such as free, glycosylated forms, ellagin, and combined forms ETs (Abe et al., 2012; Bala et al., 2006). There are several sources of EA which are mainly found in different berries such as strawberry, goji berry, and cranberry, as well as it could be found in nuts and mushrooms (Evtyugin et al., 2020). Moreover, honeybee's food supplement is a rich source of the free and glycosylated forms of EA (Zafrilla et al., 2001). EA has a low water solubility which results in poor oral bioavailability (Romo‐Vaquero et al., 2015; Saha et al., 2016). Although a small amount is absorbed into the stomach, the small intestine is the main site of EA absorption. Immediately after absorption, EA goes through metabolization and is converted to a variety of metabolites like methyl esters, dimethyl esters, or glucuronides (Bialonska et al., 2010). In the colon, the gut microbiota transfers free EA to urolithins (Uro) such as Uro‐D, Uro‐C, Uro‐A, and Uro‐B which increase its lipophilicity and enhance EA absorption (Nuñez‐Sánchez et al., 2014). More evidence has proven EA benefits for health and disease conditions like anti‐oxidant, anti‐inflammatory, and anti‐cancer properties (Duan et al., 2020; Jaman & Sayeed, 2018; Kim et al., 2021). The presence of two lactones, four hydroxyl groups, and two hydrocarbon rings enabled the EA to easily receive free electrons and act as antioxidant agents (Ríos et al., 2018). Moreover, it could clear oxygen free radicals and metal ions such as iron and copper as well as interfere with several signaling pathways (Gupta et al., 2021; Shakeri et al., 2018). Also, it has been demonstrated that EA regulates enzyme activities like glutathione peroxidase (GSH‐Px), superoxide dismutase (SOD), and catalase, resulting in a reduction in ROS (Brudno & Kochenderfer, 2018).

### 2.1 Ellagic acid derivatives

EA could be found in different forms either in free form or as derivatives, which are predominantly presented in complex polymers called ETs (Landete, 2011). There are four EA derivatives including 3,3′‐dimethyl‐4′‐*O*‐β‐d‐glucopyranosyl ellagic acid, 3,3′,4‐trimethyl‐4′‐*O‐*β‐d‐glucopyranosyl ellagic acid, 3′‐methyl‐3,4‐*O*,*O‐*methylidene ellagic acid, and 3′‐methyl‐3,4‐*O*,*O*‐methylidene‐4′‐*O‐*β‐d‐glucopyranosyl ellagic acid (Corrêa et al., 1985; Do Khac et al., 1990; Nawwar et al., 1982). According to the chemical structure of EA, there are four free OH groups, two lactones, and two hydrocarbon rings, so EA is an amphipathic chemical. The free form of EA contains 40–50 percent of the total EA in berry fruit (Wada & Ou, 2002). ETs as a combined form of EA go through hydrolysis processing with acids and bases resulting in hexahydroxydiphenic acid (HHDP). Furthermore, ETs and EA could be metabolized by colon microbiota to yield Uro‐A and Uro‐B (Quideau & Feldman, 1996).

## 3 EFFECTS OF ELLAGIC ACID ON BREAST CANCER

In recent decades, several studies continue to accumulate on the beneficial effect of EA on different diseases such as cancers. Evidence revealed that EA could significantly influence tumor growth due to its antioxidant and anti‐carcinogenic properties (Ceci et al., 2018; Vanella et al., 2013). EA not only has anti‐tumor and antioxidant properties but also has anti‐proliferative and anti‐angiogenesis activities (Chen et al., 2015; Kim et al., 2009; Yousuf et al., 2020). The anticancer activity of EA on breast mass was first characterized by Saleem et al. in 2002. They depicted that phenolic compounds had an inhibitory effect on cell proliferation and cell viability as well as induced cell death in numerous malignant cell lines (Saleem et al., 2002).

CDK6 is important for cancer progression and is responsible for the regulation of key metabolic processes and cell cycle progression (Tadesse et al., 2015; Tigan et al., 2016). CDK6 and cyclin D phosphorylate and inactivate the retinoblastoma protein (RB) (Gao et al., 2020). CDK6 overexpression has been observed in various cancers, like BC (Wolff, 2016). Its overactivity contributes to the development and progression of tumors (Hsu et al., 2014), and its suppression has been proposed as a treatment strategy for cancers (Lee & Zeidner, 2019). Yousuf et al. investigated that EA suppresses the activity of CDK6 in BC cells through its binding affinity and subsequent interference with the enzyme's function (Yousuf et al., 2020). EA hinders the proliferation of MCF‐7 human BC cells through the TGF‐β/Smad3 pathway (Zhang, Chen, et al., 2014). EA induces G0/G1 cell cycle arrest in MCF‐7 BC cells (Zhang, Chen, et al., 2014). EA has anti‐angiogenesis benefits through VEGFR‐2 signaling axis in BC (Wang, Wang, et al., 2012). It significantly inhibits VEGF‐induced angiogenesis processes such as proliferation, migration, and tube formation of endothelial cells (Wang, Wang, et al., 2012). Furthermore, it inhibits VEGFR‐2 tyrosine kinase activity, MAPK, and PI3K/Akt in endothelial cells (Wang, Wang, et al., 2012).

Overall, EA has shown promising effects on inhibiting BC cell proliferation and inducing apoptosis, as well as in inhibiting angiogenesis in BC. However, more investigations are essential to fully understand the underlying mechanisms and to determine the optimal dosage and administration of EA for BC prevention and treatment. We further describe the effects of EA on BC cells with an emphasis on cellular and molecular mechanisms.

### 3.1 Molecular mechanism of Ellagic acid

**FIGURE 2:**
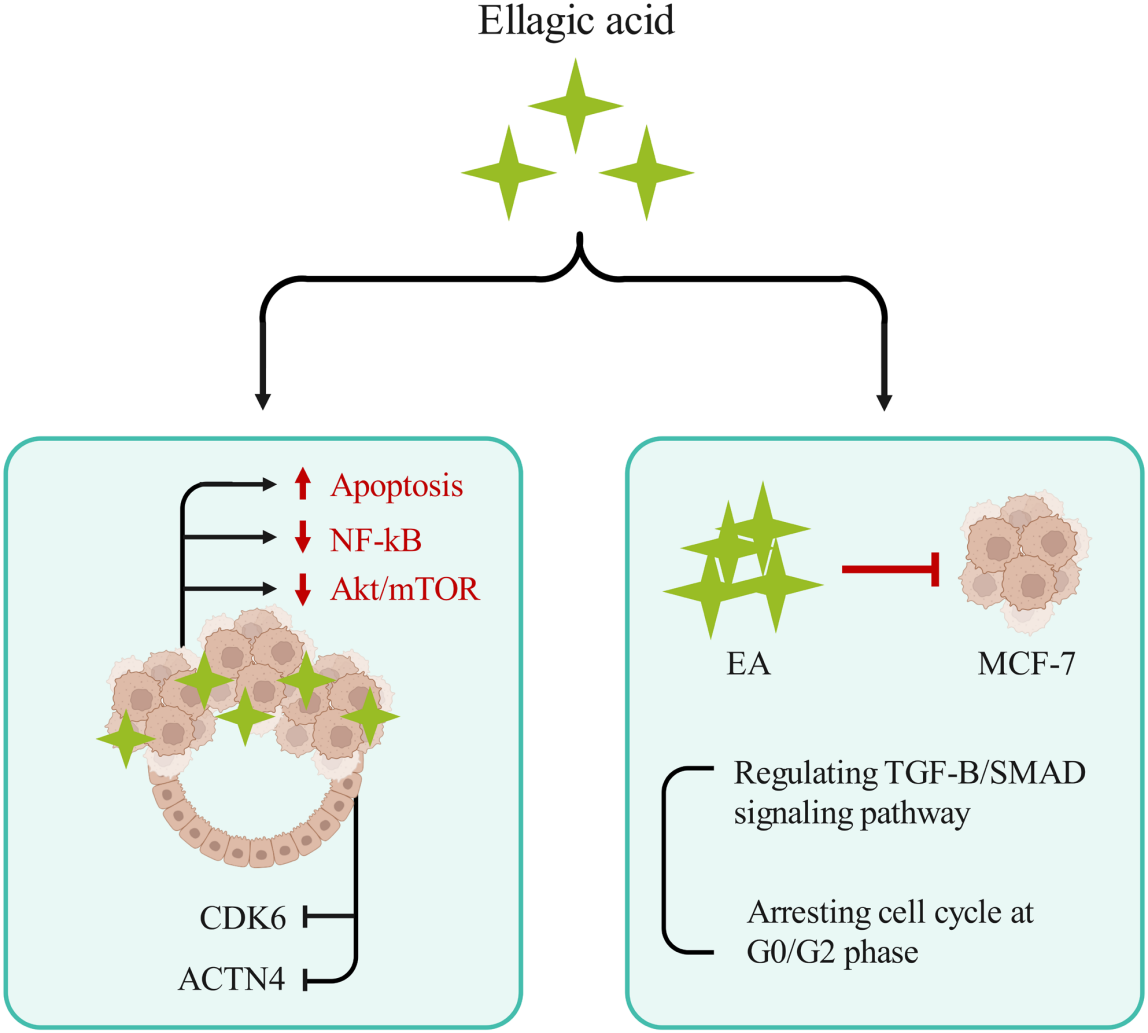
Effects of Ellagic acid on breast cancer: cellular effects and molecular mechanisms.

EA's anticancer activity is mainly associated with numerous cellular and molecular mechanisms (Figure 2). The TGF‐β/Smads signaling system is one of the well‐described pathways by which EA applies growth inhibitory effect on BC cells. This signaling system is responsible for a wide range of biological processes, such as cell growth, cell death, and angiogenesis (Ho et al., 2005; Ochoa et al., 2018). Chen et al. concluded that EA could prevent the MCF‐7 BC cell proliferation in the G0/G1 phase of the cell cycle. Furthermore, they found that the TGF‐β/Smads signaling pathway is a potential underlying mechanism that is induced by EA to control cell cycle arrest in vitro. Hence, modulating the TGF‐β/Smads signaling system in BC cells might be a promising strategy for individuals suffering from breast mass. The findings further demonstrated the downregulation of cyclins (cyclin A2 and cyclin E2) as well as the upregulation of CDK inhibitors (p21^Cip1^, p15, and p19) in EA‐treated MCF‐7 cells (Chen et al., 2015).

The CDK inhibitors P21, p27, and p15 are secondary targets of the TGF‐β signaling pathway which regulates the cell cycle. They could have an essential role in decreasing tumor growth rate (Xu & Pasche, 2007). Moreover, another study investigated the suppressing effect of EA on MCF‐7 mediated by the TGF‐β/Smads signaling system. The results showed that EA induced cell cycle arrest and inhibited cancer cell proliferation and co‐treatment with a specific inhibitor of Smad3 (SIS3) and attenuated the antitumor activity of EA (Zhang, Chen, et al., 2014).

Adam et al. study demonstrated that treatment with blueberry phytochemicals decreased the (PI3K)/AKT and nuclear factor‐kappa B (NF‐κB) activation which significantly lowered the growth and metastatic potential of MDA‐MB‐231 cells (Adams et al., 2010). Moreover, co‐treatment with EA and PI3K inhibitor GDC‐0941 (a phosphatidylinositol‐4,5‐bisphosphate 3‐kinase inhibitor) has been illustrated to drastically reduce BC cell proliferation, invasion, and migration in vitro, as well as prevent tumorigenesis in vivo. In addition, it has been observed that combination therapy increased apoptosis and decreased AKT/mTOR activity (Shi et al., 2015).

EA not only interferes with the TGF‐β/Smads and PI3K signaling pathways but is also associated with the AKT/mTOR signaling system. AKT/mTOR signaling system regulates a variety of cellular processes like cell growth, metabolic activity, angiogenesis, autophagy, and cell death and it could be suppressed via EA. Additionally, the EA exerts its effect against invasion and migration by altering the three gene expression including interleukin‐13 receptor subunit alpha‐2 (IL‐13Rα2), cyclooxygenase‐2 (COX‐2), and neural precursor cell expressed developmentally downregulated protein 9 (NEDD‐9), in all four BC cell lines (SKBr3, UM159, MDAMB‐231, and HCC1954 cells) (Shi et al., 2015). Khan et al. detected that NF‐κB precursor protein p105 is another target of EA which is overexpressed in BC. EA suppressed NF‐κB precursor protein p105 in BC cells (Khan et al., 2013). Moreover, it has been revealed that EA could suppress DNA methyl transferase 1 (DNMT1) which has a key role in the hypermethylation of tumor suppression of genes resulting in diseased conditions (Rasmi et al., 2023). CDK‐6 is one more key factor in BC due to its crucial role in the development of cancer (Yousuf et al., 2020, 2022). Yousuf et al. demonstrated that EA therapy caused a reduction in the number of cancer cells and induced apoptosis. Moreover, EA downregulated the CDK‐6 gene expression in human BC cell lines. They showed that EA attached to the ATP binding pocket of CDK6 and formed covalent connections which prompted the suppression of CDK6 expression in MDA‐MB‐231 and MCF‐7 cells (Yousuf et al., 2020). Alpha‐actinin‐4 (ACTN4) is a further therapeutic target in BC which was first discovered by Honda et al. as a factor associated with cell motility and cancer metastasis (Honda et al., 1998). While ACTN4 suppression decreased the aggressiveness of tumor cells, overexpression of ACTN4 enhanced cell motility and dissemination (Sugano et al., 2020; Yamada et al., 2010). In this regard, Wang et al. revealed the potential impact of EA on ACTN4 in vitro (MCF‐7, BT‐549) and in vivo (mice model) accompanied by small cancer stem cells (CSCs) population. The findings indicated that malignant cell growth, colony formation, and metastatic potential were all inhibited by ACTN4 suppression. Moreover, it was found that a higher frequency of metastasis, an aggressiveness of cancer, and shorter overall survival rates were all significantly correlated with increased ACTN4 activity (Wang et al., 2017).

It is widely investigated that EA could prevent pathological angiogenesis of cancers. Studies confirmed that angiogenesis can be seen in breast mass during all stages of development, proliferation, and metastasis which is associated with vascular endothelial growth factor (VEGF) as the most effective angiogenesis activator (Fox et al., 2007; Kurebayashi et al., 1999; Senger et al., 1993). Wang et al. found that EA not only interferes with VEGF‐induced angiogenesis but also inhibited VEGFR‐2. So EA is a suitable therapeutic option as an anti‐angiogenesis drug with minimal side effects (Wang, Wang, et al., 2012). Another study showed a decreased number of MCF‐7 and Hs 578 T cells as well as apoptosis induction after EA therapy by lowering ATP levels. Furthermore, the study showed a reduction in VEGF‐165, Pro‐matrix metalloproteinase‐2 (pro‐MMP‐2), and pro‐matrix metalloproteinase‐9 (pro‐MMP‐9) concentrations mediated by EA (Losso et al., 2004). ER positive is the major subtype of BC, indicating that anti‐estrogen agents might have a beneficial activity in controlling breast mass growth. It is well known that EA has an anti‐estrogenic effect by considerably increasing the concentrations of insulin‐like growth factor‐binding protein 3 (IGFBP3) in MCF‐7 cells (Papoutsi et al., 2005). Moreover, more evidence revealed the probable telomerase role in more than 85% of human cancers (Kirkpatrick & Mokbel, 2001; Shay & Bacchetti, 1997). Research showed that human telomerase reverse transcriptase (hTERT) gene expression along with two oncogenes caused direct tumor transformation of healthy cells (Hahn et al., 1999). On the other hand, Strati et al. depicted the inhibitory effect of EA on hTERT α + β + in MCF‐7 cells. These findings suggest that EA could be regarded as the chemo‐preventive agent of BC (Strati et al., 2009). Additionally, it has been discovered that 17‐estradiol (E2) stimulated hTERT activity in MCF‐7 cells via an ER‐dependent pathway (Kyo et al., 1999).

### 3.2 Drug delivery of Ellagic acid

Recent studies showed the promising results of applying nanomedicine in the diagnosis and treatment of life‐threatening disorders such as malignancies. Cancer nanomedicine can detect malignant growth with high sensitivity and improves treatment efficacy through a variety of methods, including nano‐sized substance delivery platforms and nano‐pharmaceuticals (Sabra et al., 2017; Wang et al., 2013). Also, nanotechnology could enhance the selectivity of physical, chemical, and biological techniques in tumor cell destruction while limiting the toxic consequences of non‐cancerous cells (Gmeiner & Ghosh, 2014). Moreover, extracting the drug from the nano‐carriers and embodying it inside them could solve some pharmacological restrictions such as water insolubility, poor bioavailability, enzymatic degradation of drugs, and weak bio‐distribution in the circulatory system (Arulmozhi et al., 2013; Wilczewska et al., 2012). Regarding the fact that EA has low bioavailability, Mady et al. solved this issue by using an efficient strategy. They showed that the encapsulation of EA in biodegradable polymeric nanoparticles would improve the bioavailability after oral administration and also enhance the anticancer properties (Mady & Shaker, 2017). One more study described that a combination of Chitosan nanoparticles and EA with high anticancer efficacy could be a suitable therapeutic strategy for BC. Kaur et al. compared the expected release of EA from tween 80‐coated EA‐chitosan NPs and uncoated chitosan nanoparticles which showed higher PDI. They also found that nanoparticles were more effective than EA (50 mg/kg) in terms of tumor recurrence and tumor tissue necrosis (Kaur et al., 2021). Another nanoparticle called mesoporous silica nanoparticles (MSNPs) has been established as an efficient drug carrier with excellent biocompatibility and modulation of the immune system (Luo et al., 2014). Lactoferrin (Lf)‐coupled MSNPs is a carrier of the cytotoxic drug pemetrexed (PMT) and the phytomedicine EA combination. When compared to free pharmaceuticals, the dual drug‐loaded Lf‐MSNPs showed the greatest toxicity toward MCF‐7 BC cells, as shown by the minimum mixture index (Ali et al., 2020). Apamin‐functionalized emulsomes (EA‐EML‐APA) are a further nano‐carrier that increases cytotoxic effects and apoptosis of EA against MCF‐7. They also provided information on lower cell viability, expression of apoptotic components (Bcl‐2, Bax, p53, and casp‐3), and the inhibition of NF‐B function. Moreover, EA‐EML‐APA interfered with G2/M and S cell cycle and prevented the growth of MCF‐7 cells (Badr‐Eldin et al., 2022). According to Pirzadeh et al. research, MCF‐7 was successfully suppressed by EA/schizophyllan‐NP and EA/chitin‐NP with IC50 values of 60 and 115 g/mL, respectively (Pirzadeh‐Naeeni et al., 2020).

#### 3.2.1 Combination drugs and therapy

It was suggested that a combination of EA with chemotherapy as well as radiotherapy could enhance treatment outcomes and optimize the dose of drugs and radiation. Ahire et al. demonstrated that the combination of EA (10 mM) with radiotherapy substantially increased the cytotoxicity in human BC cells (MCF‐7). A reduction in mitochondrial membrane potential (MMP) and nuclear injury was found following the treatment. Furthermore, EA facilitated the recovery of normal cells as well as induced tumor toxicity after radiation (Ahire et al., 2017). Moreover, another study depicted that EA might be a great potential drug adjuvant for cancer treatment and showed that the synergistic effect of EA combined with radiotherapy/chemotherapy resulted in increased DNA damage and apoptosis as well as decreased levels of MGMT expression. Also, due to EA's anti‐oxidant and anti‐inflammatory properties, the probable side effects of chemo‐radiotherapy would be diminished (Xue et al., 2022). Bhosle et al. found that treatment of cancer with EA followed by radiation of 2 Gy would increase oxidative stress and cytotoxicity in tumor cells. They observed decreased levels of SOD, GSH‐Px, and catalase in tumor cells (Bhosle et al., 2005). In addition, it has been detected that after exposure of Swiss Albino mice with 6 Gy of electron beam radiation and 100, 200, and 400 mg/kg body weight of pomegranate extracts and synthetic ellagic acid, the anti‐oxidative enzymes were increased (Bhandary et al., 2014).

Paclitaxel is a type of antimicrotubule agent that works by inhibiting the microtubule disassembly process, causing cell cycle arrest and apoptosis (Alli et al., 2002), and is used to treat various types of cancer, like BC (Khan et al., 2020). Paclitaxel is often used in combination with other drugs for chemotherapy in BC. Paclitaxel has been studied in combination with bevacizumab as an initial treatment for metastatic BC (Miller et al., 2007). Paclitaxel has also been studied in combination with atezolizumab in advanced triple‐negative BC (Schmid et al., 2018). The combination of Paclitaxel and EA has shown promise in inhibiting tumor growth and metastasis in experimental BC models. Transmission electron microscopic studies have provided insights into the effects of these drugs on cancer cells, including cell shrinkage, nuclear condensation, and fragmentation (Jamunakumari & Sakthisekaran, 2014). Another study showed that EA has a synergistic effect in combination with PI3K inhibitor GDC‐0941 in BC. The combination of EA and GDC‐0941 notably suppressed cell growth under attached and detached conditions and inhibited migration and invasion in vitro as well as tumor initiation and metastasis in vivo. Additionally, EA induced apoptosis and decreased the activity of AKT/mTOR in GDC‐0941‐treated BC cells (Shi et al., 2015).

#### 3.2.2 Toxicological studies

In comparison with synthetic drugs, herbal agents provide greater choice for patients due to their easy access and cost‐effective properties (Kamboj, 2000). Several studies revealed the beneficial effects of plant derivatives on the treatment of various diseases. Previous reports indicated some toxic outcomes of herbal medicine in different organs including kidneys, liver, heart, and brain (Au, 2006; Ernst, 2003; Gaibazzi et al., 2002; Kaplowitz, 1997; Saad et al., 2006). Despite the promising results of EA in cancer management, the safety assessment of EA is an important concern. Hurtado‐Nuñez et al. investigated that gallic and ellagic acid induced dose‐dependent renal and cardiac toxicity following uncontrolled use at high doses (≥200 mg/kg) (Hurtado‐Nuñez et al., 2022). Moreover, another article depicted that 630 mg/kg is the LD_50_ of EA in the rat population. They found reduced body weight in female rats following intraperitoneal administration of EA (Tasaki et al., 2008). Bhandary et al.'s research is another piece of evidence that detected that EA had no deleterious effect on body organs and the no‐observed adverse effect level of EA is 2000 mg/kg body weight (Bhandary et al., 2013). Heilman et al. aimed to assess the safety of Uro‐A, an EA metabolite, in terms of genotoxicity, toxicokinetics, and repeated oral dose toxicity of synthetic Uro‐A. They observed no organ toxicity or changes in biochemistry profiles (Heilman et al., 2017). Currently, no adverse effects have been observed in association with EA in human populations either consumed as a supplement or as a part of the diet (Muthukumaran et al., 2017). Numerous research showed the advantageous role of EA against a variety of cancers including bladder, breast, colon, and pancreatic cancer and found EA as a non‐toxic and safe agent (Aiyer et al., 2008; Ceci et al., 2016; Cheng et al., 2017; Naiki‐Ito et al., 2015; Shi et al., 2015; Umesalma et al., 2014). Furthermore, Zhao et al. illustrated the inhibitory activity of EA on pancreatic tumor cell growth, metastasis, and angiogenesis. They concluded that EA could safely prevent and treat different cancers (Zhao et al., 2013). Also, more evidence proposes the protective role of EA in many body tissues such as neurons, the liver, and kidneys (Afifi et al., 2018; Chen et al., 2022; Javaid et al., 2021).

## 4 CONCLUSION

EA is a natural phenolic agent that has been reported to possess multiple biological effects, including antioxidant, anti‐inflammatory, and anticancer activities. Several articles depicted that EA could block the growth and proliferation of BC cell lines like MCF‐7, MDA‐MB‐231, and BT‐474 cells. EA promotes apoptosis in BC cells via different mechanisms. This includes activation of the p53 tumor suppressor pathway, downregulation of anti‐apoptotic Bcl‐2 proteins, and inducing cell cycle arrest. EA stops the growth and proliferation of BC cells by inhibiting G0/G1 cells.

It has been found to suppress angiogenesis needed for tumor growth and metastasis. EA blocks VEGF, an important protein involved in angiogenesis. EA exhibits dose‐dependent cytotoxicity, meaning higher concentrations exhibit more potent anticancer effects. However, it demonstrates selectivity for cancerous cells over normal breast epithelial cells. Combining EA with chemotherapeutic drugs like doxorubicin shows synergistic effects, enhancing each other's anticancer actions at lower doses compared to individual drugs. EA specifically interferes with several signaling cascades, including PI3K/AKT, NF‐B, CDK6, TGF‐/Smad3, and AKT/mTOR, to perform its anti‐tumor actions. Animal studies also showed that EA suppressed tumor growth and stimulated apoptosis in BC xenograft models, without apparent toxicity. Additionally, both in vitro and in vivo EA nanoparticles have notably increased their cytotoxic effects. EA is a natural substance that showed promising anticancer properties when used as adjuvant therapy (Table 1).

**TABLE 1:** Studies consist of the purpose of the present review.

Authors	Dosage of EA	Type of study/Model	Mechanisms of EA
Yousuf et al.	3.053 μM	MCF‐7 and MDA‐MB‐231 cells	Inhibits CDK6 activity in BC cells through its binding affinity to CDK6
Chen et al.	2 mg/mL	MCF‐7 breast carcinoma cells	Downregulation of cyclin A2 and cyclin E2. CDK inhibitors (p21Cip1, p15, and p19) increase
Wang et al.	25 M	Animal study/Mice	Inhibition of BC growth and metastasis via directly targeting ACTN4 in vitro and in vivo
Ahire et al.	10 mM	Breast carcinoma cells	Upregulation of pro‐apoptotic Bax and downregulation of Bcl‐2
Kaur et al.	50 mg/kg	MCF‐7 breast carcinoma cells	Induced higher apoptosis in tumor tissues
Strati et al.	10(−)(9) M‐10(−5) M	MCF‐7 breast carcinoma cells	Reduction of 17β‐estradiol and increase of hTERT alpha+beta+ mRNA expression
Shi et al.	100 mg/kg/day	SUM159, HCC1954, MDA‐MB‐231, SKBR3, and 4 T1/mice	Promotes apoptosis in GDC‐0941‐treated BC cells/reduces tumor growth and nearly complete inhibition of metastatic spread
Wang et al.	2.5–20 μM	MDA‐MB‐231, SKBR3, HCC1954 and 4 T1	Inhibits VEGF‐induced angiogenesis processes in BC through multiple mechanisms such as MAPK and PI3K/Akt
Zhang et al.	Concentration‐dependent manner	MCF‐7	Blocks the proliferation of MCF‐7 BC cells through modulation of the TGF‐β/Smad3 signaling axis

## AUTHOR CONTRIBUTIONS

**Sahbanathul Missiriya Jalal:** Data curation (equal). **Sara Abdalrazzaq M. Noraldeen:** Data curation (equal). **Andrés Alexis Ramírez‐Coronel:** Resources (equal). **Khulood H. Oudaha:** Resources (equal). **Rasha Fadhel Obaid:** Methodology (equal); resources (equal). **Abbas F. Almulla:** Data curation (equal); resources (equal). **Gholamreza Bazmandegan:** Validation (equal); writing – original draft (equal). **Zahra Kamiab:** Conceptualization (equal); project administration (equal); validation (equal); visualization (equal); writing – review and editing (equal).

## FUNDING INFORMATION

This research received no external funding.

## CONFLICT OF INTEREST STATEMENT

The authors declare no conflict of interest.

## ETHICS STATEMENT

This article does not contain any studies with human participants or animals performed by any of the authors.

## Data Availability

The data relevant to the review article are within the manuscript.
